# Pulmonary arteriovenous malformation in a child with multiple brain abscesses: A case report

**DOI:** 10.7196/AJTCCM.2021.v27i1.109

**Published:** 2021-03-09

**Authors:** S T Hlophe, R Masekela

**Affiliations:** Department of Paediatrics and Child Health, Nelson R Mandela School of Clinical Medicine, University of KwaZulu-Natal, Durban, South Africa

**Keywords:** pulmonary, arteriovenous malformation

## Abstract

Pulmonary arteriovenous malformations (PAVMs) are caused by abnormal connections between arteries and veins, which lead to right-to-left shunting of deoxygenated blood. Here, we report an 11-year-old male who presented with signs suggestive of intracranial pathology. The patient displayed signs of a chronic illness, possibly congenital malformation that was complicated by PAVM and multiple brain abscesses. This case illustrates the importance of doing a detailed examination and investigations, especially if the history alone is not helpful in making a diagnosis.

## Case

An 11-year-old male presented to the local clinic with a headache for 2 weeks and he was given analgesia. He later developed eye pain and extreme photophobia, which led to a visit to a general practitioner where influenza was diagnosed. The symptoms did not resolve and he presented 10 days later to the clinic with difficulty breathing, worsening headache, weakness of the lower limbs and inability to walk. He was referred to the nearest hospital. The mother reported that he had acute respiratory tract infections since the age of 9 years, which resolved spontaneously and he had never been to a healthcare facility for management of the respiratory complaints. There was no family history of note. Both siblings were well with no chronic illnesses. He was examined and found to have cyanosis, clubbing and proptosis of both eyes. His oxygen saturation fluctuated between 60% and 82% pre- and post-ductal, tachycardia was at 170 bpm and he was hypotensive (84/43 mmHg), with a delayed capillary refill time of >3 seconds. Cardiovascular examination revealed normal heart sounds with a murmur heard on the left lateral aspect of the chest. Other than hypoxia, there were no noteworthy findings in his respiratory system. Central nervous system examination revealed signs of upper motor neuron lesions on the left side

**Fig. 1:**
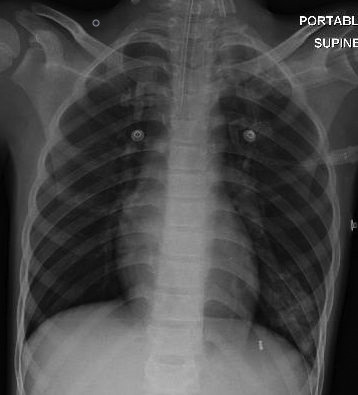
X-ray of the chest showing opacification on the left lower zone.

Full blood count showed high white cell count of 27.9 × 10^9^ /L, haemoglobin was 16.9 g/dL and the number of platelets was 344 × 10^9^/L. Electrolytes analyses showed that the levels of sodium were 128 mmol/L, potassium was 4.6 mmol/L, chloride was 93 mmol/L, bicarbonate was 16 mmol/L, urea was 2.9 mmol/L and creatinine was 24 mmol/L. Creatine kinase levels were elevated at 1 011 U/L. Blood gas analyses revealed that the pH was 7.39, partial pressure of oxygen (PaO_2_) was 48 mmHg, PaCO_2_ was 49 mmHg, bicarbonate ion was 30 mmol/L and base excess was 5 mmol/L. A lumbar puncture showed high protein at 4.5 g/dL, low chloride at 110 mmol/L and cell count was not done. Chest X-ray showed opacity on the left side (Fig. 1).

**Fig. 2:**
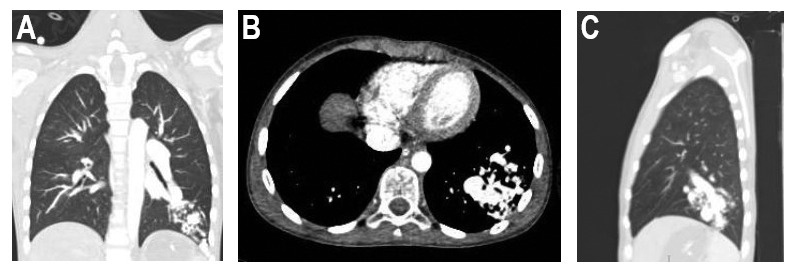
Computed tomography chest image showing pulmonary arteriovenous malformations in the left lower lobe. Posterior-anterior view (A), transverse view (B), and lateral view (C).

**Fig. 3:**
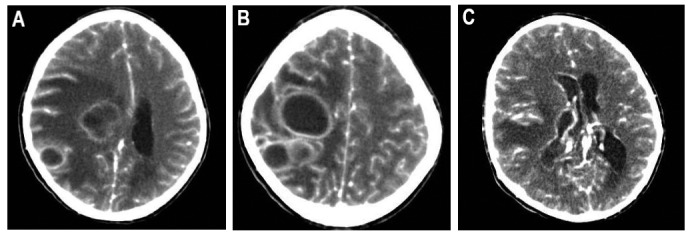
Computed tomography brain image showing rim-enhancing collections in the right frontal (A) and parietal lobe with mass effect (B). Computed tomography brain image showing diffuse enhancement of the ependyma of the right lateral ventricle (C).

Echocardiogram showed a structurally normal heart, dilated inferior vena cava and a hyperdynamic myocardium. Further investigations included computed tomography (CT) of the chest and brain. The CT angiogram showed large left lower-lobe pulmonary arteriovenous malformation (PAVM) and intracranial infective processes that included abscesses, ventriculitis and meningitis with significant mass effect and intracranial herniation. Bilateral cerebellar infarcts were also noted (Figs 2 and 3) .

Management of the patient included mechanical ventilation for hypoxia and decreased level of consciousness, vasopressor therapy for cardiogenic shock, antibiotics for the suspected meningitis, immunoglobulins for suspected inflammatory myositis and counselling for the mother regarding the clinical condition and the possible diagnosis.

The plan was to transfer the patient to a quaternary hospital for further management that included intracranial abscess drainage. A bed was not available at the time of initial discussion. The patient deteriorated 2 days later, developed diabetes insipidus (serum sodium 189 mmol/L, serum osmolality 369 mmol/L and urine osmolality of 88 mmol/L) and desmopressin was administered. Brainstem test was performed and it confirmed that the patient was brain-dead. The patient demised on the ventilator.

## Discussion

PAVMs are caused by abnormal communication between pulmonary arteries and veins.^[1]^ PAVMs are fairly uncommon and the exact incidence in the paediatric population is unknown.^[1]^ Hereditary haemorrhagic telangiectasia (HHT) is the most common aetiology for PAVM in children.^[1]^ HHT is an autosomal dominant disease with multi-systemic vascular dysplasia. A tenth of patients with HHT suffer major disability or die prematurely, primarily as a result of PAVMs and cerebral AVMs.^[2]^ Simple PAVMs are defined as one or more afferent feeding arteries originating from a single segmental pulmonary artery, and complex PAVMs are characterised by multiple afferent feeding arteries originating from several segmental arteries.^[3]^ HHT is characterised by mucocutaneous telangiectasia and arteriovenous malformations (AVMs) in several organs, but primarily affecting the lungs, gastrointestinal tract and brain.^[3]^ It presents with recurrent epistaxis, telangiectasia, visceral AVMs and a first-degree family member with HHT.^[3]^ Most PAVMs are congenital and are due to HHT.^[4]^

Children who present with shortness of breath, exercise intolerance, clubbing, cyanosis and haemoptysis may be diagnosed with PAVMs. However, more than half (56%) of children diagnosed with PAVMs may be asymptomatic at the time of detection,^[2]^ suggesting that relying on clinical findings alone may not identify children at risk of significant morbidity.^[3]^ Less than one-third of affected individuals exhibit physical signs indicating a substantial right-to-left shunt (e.g. cyanosis, clubbing, and polycythaemia).^[4]^

Chest X-ray, bubble echocardiogram, CT angiogram and magnetic resonance angiogram are effective tools for identification of PAVMs, confirmation and classification according to angioarchitecture (simple and complex).^[5]^ CT angiogram is generally considered the gold standard investigation for diagnosing PAVMs, demonstrating their size and extent before therapy.^[5]^

Most PAVMs remain stable in size. However, ~25% will enlarge slowly at a rate of 0.3 - 2.0 mm/year.^[3]^ Macroscopic PAVMs (i.e. those seen on chest CT) are the most common AVMs seen in patients with HHT and have been associated with debilitating and life-threatening complications such as stroke, cerebral abscess, massive haemoptysis and haemothorax.^[3,5]^ Haemoptysis and haemothorax due to PAVM rupture are uncommon complications but are dramatic when they occur.^[5]^ Large PAVMs may also result in hypoxia.

Not all PAVMs require intervention. PAVM embolisation is recommended as first-line treatment of PAVMs amenable to treatment,^[4]^ and more than 99% can be successfully treated with this therapy.^[4]^ Lifelong follow-up, which includes CT angiogram, is important to assess for recanalisation and collateralisation that may occur after embolisation therapy.^[6]^ A number of adjunctive therapies can be used in the management of disease and include oxygen supplementation, venesection and antibiotic prophylaxis for surgical procedure.^[4]^

Surgical resection of the arteriovenous malformation may be indicated in strictly selected cases.^[5]^ Lung transplantation has been performed for patients with PAVMs but is rarely indicated because of the short life expectancy in severely hypoxaemic patients.^[5]^

## Conclusion

PAVMs are caused by abnormal communications between pulmonary arteries and veins. Up to two-thirds of children with PAVMs are asymptomatic. Any patient with unexplained hypoxaemia should be screened for PAVMs, as symptoms are nonspecific. CT angiogram is the gold standard investigation. Instituting early management plans helps to prevent complications. Brain abscesses are rare complications seen in patients with PAVMs.
